# CD36 Senses Dietary Lipids and Regulates Lipids Homeostasis in the Intestine

**DOI:** 10.3389/fphys.2021.669279

**Published:** 2021-04-28

**Authors:** Lei Zhao, Yuqi Li, Qiuying Ding, Yanping Li, Yaxi Chen, Xiong Z. Ruan

**Affiliations:** ^1^Centre for Lipid Research, Key Laboratory of Molecular Biology for Infectious Diseases, Ministry of Education, Department of Infectious Diseases, Institute for Viral Hepatitis, The Second Affiliated Hospital, Chongqing Medical University, Chongqing, China; ^2^National Clinical Research Center for Aging and Medicine, Huashan Hospital, Fudan University, Shanghai, China; ^3^John Moorhead Research Laboratory, Centre for Nephrology, University College London Medical School, Royal Free Campus, University College London, London, United Kingdom

**Keywords:** CD36, dietary lipid, intestine, lipid homeostasis, intestinal hormones

## Abstract

Dietary lipids absorbed in the intestine are closely related to the development of metabolic syndrome. CD36 is a multi-functional scavenger receptor with multiple ligands, which plays important roles in developing hyperlipidemia, insulin resistance, and metabolic syndrome. In the intestine, CD36 is abundant on the brush border membrane of the enterocytes mainly localized in proximal intestine. This review recapitulates the update and current advances on the importance of intestinal CD36 in sensing dietary lipids and regulating intestinal lipids uptake, synthesis and transport, and regulating intestinal hormones secretion. However, further studies are still needed to demonstrate the complex interactions between intestinal CD36 and dietary lipids, as well as its importance in diet associated metabolic syndrome.

## Introduction

Diet associated metabolic syndrome (MetS) has become a global public health problem. MetS is a cluster of metabolic disorders that include abdominal obesity, dyslipidemia, hypertension, elevated blood glucose, and liver steatosis. The MetS greatly increases the risk of heart disease, stroke, diabetes, non-alcoholic fatty liver disease (NAFLD), and all-cause mortality. A positive correlation has been described between the risk of MetS and dietary lipid content. Since about 95% of dietary lipids are absorbed in the small intestine, the small intestine could play an essential role in MetS etiology.

CD36, a highly glycosylated transmembrane protein also known as fatty acid (FA) translocase (FAT), platelet GPIV, GP88, and scavenger receptor class B type 2 (SR-B2), is a multi-functional scavenger receptor with multiple ligands (Chen et al., 2019).

In humans, variants in the CD36 gene have been associated with lipid and glucose metabolism abnormality and altered susceptibility to metabolic syndrome and diabetes-associated coronary disease (Love-Gregory et al., 2016). Moreover, CD36 deficient is the only genetic deficiency state among scavenger receptors in humans. Since a patient with CD36 deficiency was found in Japan in the 1990s, numerous studies have reported that the patients with CD36 deficiency had the typical metabolic features of MetS, such as dyslipidemia, including postprandial hypertriglyceridemia, insulin resistance, and hypertension (Love-Gregory and Abumrad, 2011). These phenotypes have also been observed in CD36 knockout (CD36KO) mice (Shu et al., 2020). In addition, one strain of spontaneously hypertensive rats lacks CD36 and shows metabolic phenotypes of insulin resistance and high free fatty acid (FFA) levels, which are ameliorated by the transgenic overexpression of CD36 (Aitman et al., 1999; Pravenec et al., 2001). Here we will review recent findings on the importance of CD36 in sensing dietary lipids and regulating lipids uptake, synthesis, and transport in the intestine.

### CD36 Gene, Structure, Function and Distribution

The human CD36 gene is located on the long arm of chromosome 7 (7q21.11). The gene is ∼46 kb long and includes 17 exons and 18 introns. At least 23 alternative transcripts are known for CD36, yielding different transcripts in different tissue types (Pietka et al., 2014). There are four protein isoforms generated by alternative splicing of the CD36 gene^1^.

Human CD36 contains ∼472 amino acids, which contains two transmembrane domains, a large extracellular region containing ligand-binding sites, and a short cytoplasmic tail at the N-terminal and C-terminal. The extracellular domain of CD36 forms two hydrophobic cavities that mediate the uptake of hydrophobic molecules such as FA, cholesterol, and phospholipids. CD36 also harbors a CD36, LIMP-2, Emp sequence homologous (CLESH) domain that can interact with thrombospond-1 (TSP1) repeat 2 (TSR2) domain of TSP1 (Klenotic et al., 2013). Moreover, in CD36, the lysine-cluster region can bind to negatively charged ligands, such as oxidized low-density lipoprotein (ox-LDL), apoptotic cells, and advanced oxidation protein products (AOPPs). With the interaction with these multiple ligands, CD36 plays essential roles in regulating lipids metabolism, angiogenesis, adhesion, apoptosis and inflammation/immune response (Zhao et al., 2018a).

CD36 undergoes multiple post-translational modifications, including phosphorylation, glycosylation, palmitoylation, acetylation, ubiquitylation, and disulfide bonding. These modifications control CD36 expression, maturation and subcellular localization in cells (Luiken et al., 2016).

CD36 is expressed in multiple cell types, including platelets, monocytes/macrophages, intestinal epithelial cells, microvascular endothelial cells, smooth muscle cells, adipose tissues, skeletal muscles, and cardiomyocytes. In the intestine, CD36 is highly expressed on the brush border membrane of the enterocytes mainly localized in the proximal intestine (duodenum and jejunum). It seems to be little or absent in the ileum and colon, whereas it presents in blood vessels throughout the intestine (Cifarelli et al., 2017). It is demonstrated that CD36 is important in mediating the uptake of long-chain FA (LCFA) in skeletal muscle and adipose tissues (Pietka et al., 2012). However, the role of CD36 in the intestine is more complex.

### The Role of Intestinal CD36 in Sensing Dietary Lipids

#### CD36 Regulates Dietary Fat Intake

In humans, the CD36 gene polymorphism, which causes a decrease of CD36 protein expression, is directly associated with the capability to detect dietary FA and the preference for fat-rich diet (Keller et al., 2012; Pepino et al., 2012; Fujii et al., 2019; Bajit et al., 2020). Inhibition of CD36 on ventromedial hypothalamus (VMH) neurons leads to insensitivity of neurons to FA loadings, which may cause more food intake and body weight gain, development of fatty liver and insulin resistance in rats, suggesting CD36 is a critical molecular in both neuronal FA sensing and the regulation of metabolic homeostasis (Le Foll et al., 2015). On the taste bud cells (TBC) in the tongue, CD36 binding to dietary FA launches the signal transmission to the central nervous system producing fat taste perception and cephalic phase secretion of insulin and bile acids (Laugerette et al., 2005; El-Yassimi et al., 2008). It is also reported that the defect of CD36, both in humans and animal models decreases the release of FA-induced Ca^2+^ signaling and serotonin in TBC, which may promote more fat intake in these subjects (Ozdener et al., 2014). On the enterocytes of the proximal small intestine, CD36 mediates the conversion of diet-derived FA into a cellular lipid messenger oleoylethanolamide (OEA), which consequently activates peroxisome proliferator activated receptor alpha (PPARα) to prolong across-meal satiety (Schwartz et al., 2008). Accordingly, the prolonging meal interval induced by a lipid emulsion was absent in CD36 null mutants, indicating that CD36 may regulate the fat intake via modulating OEA production. Thus these findings provide evidence supporting that CD36 may act as a dietary FA sensor to regulate dietary fat intake and lipid homeostasis.

#### CD36 Regulates Intestinal Hormones

In addition to the absorption of dietary nutrients, the intestine is well-recognized as a virtual organ with endocrine functions. Although enteroendocrine cells (EECs) account for a small population of intestinal epithelial cells (Haber et al., 2017), they produce and release several kinds of intestinal peptides and hormones (Gribble and Reimann, 2016), including cholecystokinin (CCK) secreted by I cells, secretin by S cells, glucose-dependent insulinotropic polypeptide (GIP) by K cells and glucagon-like peptide-1 (GLP-1) by L cells. These intestinal hormones widely participate in nutrients digestion and regulation of energy balance. CCK regulates gallbladder contraction, gastrointestinal secretions, and promotes fat absorption (Kato et al., 2021). Secretin regulates the pH of the intestinal contents by stimulating water and bicarbonate secretion, and it can also synergize with CCK to induce pancreatic secretions (Afroze et al., 2013). GLP-1 and GIP are identified as incretins which enhance insulin secretion in β-cells, decrease blood glucose, and regulate nutrient absorption (Holst, 2007; Sandoval and D’Alessio, 2015; Campbell, 2020). In addition, all the peptides inhibit gastrointestinal motility and promote satiety, contributing to the regulation of fat intake (Holst, 2007; Cheng et al., 2011; Dockray, 2012; Wang et al., 2015).

CD36 has been found to be expressed on the membrane of EECs. Recent studies have demonstrated its important role in regulating the secretion of multiple intestinal hormones in response to dietary lipid loading (Lobo et al., 2001; Sundaresan et al., 2013; Little et al., 2014). CD36 regulates the release of CCK by activating cAMP/CaM-KII, and it mediates secretin release via activating cAMP-PKA pathway (Sundaresan et al., 2013). *In vivo* and *in vitro*, CD36 deficiency significantly decreases the release of CCK and secretin both in the fasted and oil-loaded state (Sundaresan et al., 2013). CD36 has also been reported to interact with incretins, including GLPs and GIP. The secretion of GLP-1 and GIP to a high-fat meal was significantly reduced in human subjects carrying CD36 rs3211938 (G/T) which decreases CD36 level by approximately 50% (Shibao et al., 2018). In the meantime, the secretion of ghrelin, a gastric peptide regulating growth and energy balance, was unchanged in rs3211938 subjects (Shibao et al., 2018), suggesting that CD36 may not regulates the secretion of ghrelin.

**FIGURE 1:**
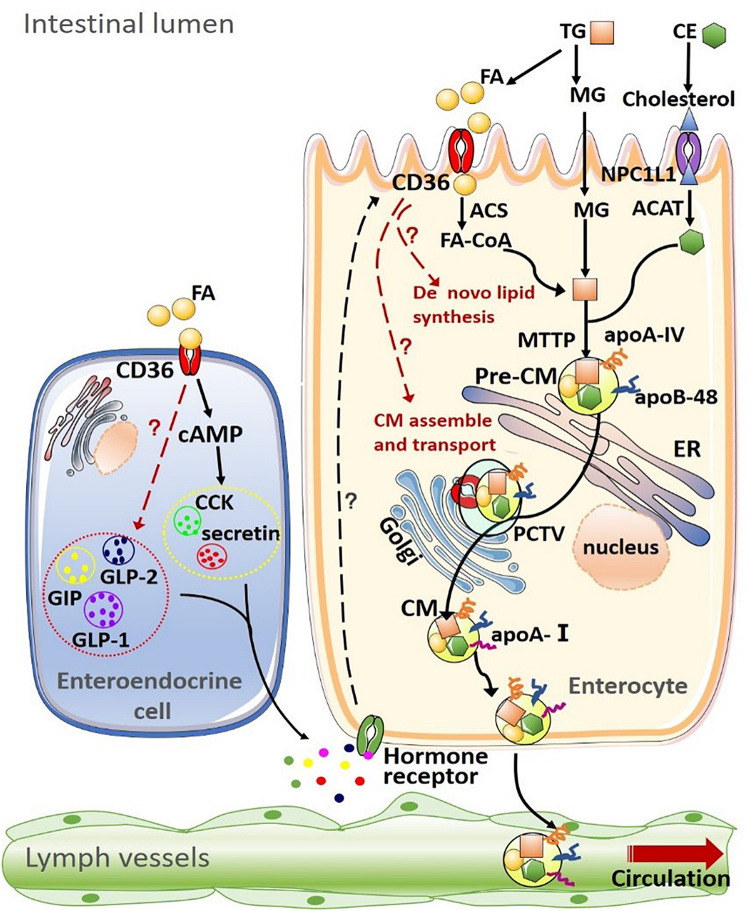
Summary of CD36 on lipid uptake, *de novo* synthesis transport and intestinal hormone in the intestine. (1) Dietary lipids, such as triglyceride (TG) and cholesteryl esters (CE) are completely digested by pancreatic enzymes in the intestinal lumen, producing fatty acids (FAs), monoacylglycerols (MAGs), cholesterol. The uptake of FAs and MAGs into the enterocyte can be driven by the concentration gradient or facilitated by other proteins such as CD36. Cholesterol uptake is mediated by Niemann-Pick C1-like 1 (NPC1L1) protein. After being taken up by enterocytes, products of lipid digestion are re-esterified by a well-coordinated group of proteins [including acyl-CoA synthetase (ACS) and acyl-CoA:cholesterol acyltransferase (ACAT)] and assembled into TG and CE. Intestinal lipids are then packaged into pre-chylomicrons (pre-CM) with apolipoprotein A-IV (apoA-IV) and apolipoprotein B-48 (apoB-48) or stored as intracellular lipid droplets. Microsomal triglyceride transfer protein (MTP) mediates the packaging of lipids into pre-CM, which are transported from the endoplasmic reticulum to the Golgi apparatus for maturation via the pre-chylomicron transport vesicle (PCTV). Mature chylomicrons (CM) are then secreted into intestinal lymph trunk and transported by the lymphatic system into the circulation. (2) CD36 on the enteroendocrine cell (EEC) mediates the secretion of multiple intestinal hormones, including cholecystokinin (CCK), secretin, glucose-dependent insulinotropic polypeptide (GIP), glucagon-like peptide-1 (GLP-1), and glucagon-like peptide-1 (GLP-2). In turn, these intestinal peptides/hormones act on their receptors on enterocytes to modulate CD36 expression and function.

In turn, the intestinal peptides/hormones can regulate the expression and function of intestinal CD36 (Little et al., 2014). Secretin and CCK can act on their receptors on enterocytes to up-regulate CD36 expression, promoting intestinal lipid absorption (Sekar and Chow, 2014; Demenis et al., 2017). Consistently, the CD36 levels were reduced in secretin receptor-deficient mice (Sekar and Chow, 2014). GLP-1 inhibits the expression of CD36 via activating protein kinase A (PKA) in human macrophages and rodents’ cardiomyocytes (Dai et al., 2014; Wu et al., 2018). Interestingly, GLP-2, a product of the same gene *gcg* as GLP-1, promotes lipid absorption by increasing the expression of fully glycosylated CD36 in the small intestine, which involves the intestinal-epithelial insulin-like growth factor-1 receptor (Hsieh et al., 2009; Mellitzer and Gradwohl, 2011; Xiao et al., 2015; Markovic et al., 2020; Figure 1).

### The Role of CD36 in Regulating Lipids Homeostasis in the Intestine

#### CD36 in the Absorption of Dietary Triglycerides/FA

Lipid absorption in the intestine involves the digestion and absorption of triglycerides (TG), phospholipids, cholesterol esters, and fat-soluble vitamins. TG are the majority of dietary lipids, which account for about 90% of dietary lipids. In the lumen of duodenum, dietary TG is hydrolyzed into FA, mostly LCFA (16∼20 carbon atoms), monoglyceride (MG) and glycerol. The short-chain FA, medium-chain FA and glycerol can be directly absorbed into the portal system, whereas LCFA and very-long-chain FA need FA transporters to be absorbed and transported to the bloodstream.

CD36 is one of the important proteins involved in facilitating the absorption of LCFA. In the intestine, CD36 expression is abundant in the apical membrane of enterocytes in the duodenum and jejunum, the main sites of lipids absorption. Goudriaan et al. (2002) determined the TG absorption in CD36KO mice by administering an intragastric olive oil bolus with ^3^H-labeled triolein and the amount of ^3^H label in plasma, after blocking serum lipoprotein clearance by i.v. injections of Triton WR 1339. The plasma appearance of ^3^H-label in CD36KO mice is not different from that in wild-type (WT) mice. These results suggest that CD36 deficiency does not affect intestinal TG absorption after an acute lipid load. Drover et al. (2005) further monitored the TG absorption in a high-fat diet-fed mice model and showed that fecal TG levels were comparable in CD36-deficient mice and WT controls. Also, using the sucrose polybehenate method, which evaluates overall TG absorption from the ratio of fecal fat to a non-absorbable marker, it showed that no significant difference was observed between CD36KO and WT mice (Drover et al., 2005). These data indicate no global defect in overall TG absorption in CD36-deficient mice.

On the other hand, Nassir et al. (2007) isolated enterocytes from the proximal and distal small intestine of CD36KO and WT mice. Compared with WT mice, the enterocytes of the proximal intestine from CD36KO mice exhibit 50% reduction of the uptake of [^3^H]-oleate-BSA (C18:1). The mass spectrometry technique of shotgun lipidomics also shows that the oleic acid enrichment of mucosal lipids is delayed in the proximal intestine of CD36 KO mice, compared with WT mice, after gavage with olive oil. These data support that CD36 is important for LCFA uptake in the proximal intestine. Interestingly, *in vivo*, it is reported that the absorption of ^14^C-labeled palmitic acid is comparable between CD36KO mice and WT mice after gavage of olive oil with ^14^C-labeled palmitic acid (Goudriaan et al., 2002). One possibility could be that the LCFA absorption is enhanced in the distal intestine in compensation to the reduced LCFA uptake in the proximal intestine of CD36KO mice, which may explain comparable TG absorption between CD36KO and WT mice. It is also possible that other intestinal FA transporters, including fatty acid transport proteins (FATPs) and fatty acid binding proteins (FABPs) could compensate for reduced LCFA uptake in the absence of CD36 (Chassen et al., 2018). However, these hypotheses need to be validated in future works using mice models with the deficiency of these genes in the intestine.

Moreover, recent studies show that the absorption of C24:0 FA is completely abolished in intestine of CD36KO mice fed a high-fat diet (Drover et al., 2008), highlights the key role of intestinal CD36 in the absorption of fatty acids containing very long saturated acyl chains.

#### CD36 in the Absorption of Dietary Cholesterol

Using the fecal dual-isotope method, it is demonstrated that there is no significant difference in cholesterol absorption between CD36KO and WT mice 24-h after gavage with ^14^C-cholesterol and ^3^H-sitosterol (Nauli et al., 2006). However, when mice were intraduodenally infused with a lipid emulsion containing [^3^H] triolein and [^14^C] cholesterol, compared with WT mice, the CD36KO mice exhibited significant accumulation of recovered [^14^C] cholesterol in the small intestinal lumen and significant reduction of recovered [^14^C] cholesterol in the lymph at the end of 6-h infusion (Nauli et al., 2006). It seems that while CD36 deficiency reduced the absorption of dietary cholesterol in a short time, it does not affect global dietary cholesterol absorption for a long time.

Using primary enterocytes, it is also demonstrated that CD36 deficiency reduces cholesterol uptake (60%) in proximal intestine but not in the distal intestine (Nassir et al., 2007). Thus it is suggested that CD36 mediates the cholesterol absorption in the intestine, but its absence can be compensated by many factors, including slower gastrointestinal emptying, a longer length of small intestine and the presence of other cholesterol transporters. Consistently, it is demonstrated that CD36KO mice exhibits significantly higher levels of Niemann-Pick C1 Like 1 (NPC1L1) in the distal intestine compared with WT mice (Nassir et al., 2007). Since NPC1L1 is a key molecule to the cholesterol absorption in the intestine (Zhang et al., 2018), it is proposed that intestinal CD36 may also be involved in the regulation of cholesterol absorption via NPC1L1 pathway.

#### CD36 Regulates *de novo* Lipogenesis and Lipids Transport in the Intestine

Once FA is taken up by binding proteins/transporters at the apical surface of enterocytes, it can be converted to the corresponding acyl-CoA, which is rapidly resynthesized into TG. This resynthesized TG together with endogenous TG are packaged with apolipoproteins (apo) (apoA and apoB-48) into chylomicrons (CM) (Nauli et al., 2006) in the ER of intestinal mucosal cells. They are then transported to the basolateral side of enterocytes and secreted into the extracellular space of intestinal epithelial cells, collected by lymph. Lymph goes through thoracic duct and into left brachiocephalic vein and blood. In the blood, it gets apo C and E from high-density lipoprotein (HDL).

In patients with CD36-deficiency, TG levels in very low-density lipoprotein (VLDL) and low-density lipoprotein (LDL) fractions are higher than controls in fasting state. After oral fat loading (OFL), the levels of TG in CM, VLDL, and LDL are enhanced further in the patients with CD36 deficiency compared with controls. The TG concentrations of apoB-48-containing fractions are also higher in the patients than controls, during fasting and postprandial state (Masuda et al., 2009). These increases in TG, apoB-48, FFA, and free glycerol after OFL suggest that CD36 might be involved in the production and/or transport of lipids of intestine in response to fat loading.

In animal models, Masuda et al. (2009) showed that the TG, FFA and free glycerol levels in the intestinal lymph of CD36-KO mice were significantly increased after gavage of olive oil compared with WT mice. Interestingly, Drover et al. (2005) and Goudriaan et al. (2002) showed that the recovery of [^3^H]-labeled TG infused into the duodenum was reduced in the intestinal lymph of CD36KO mice compared with WT mice (Nauli et al., 2006). It is known that the intestinal TG is derived from both exogenous and endogenous sources, and the endogenously synthesized intestinal TG accounts for more than half of total lymphatic TG during the postprandial state (Shiau et al., 1985). Thus, these data suggest that while the exogenously synthesized TG derived from dietary source is decreased in the intestine of CD36KO mice, the endogenously synthesized intestinal TG is increased, contributing to an enhanced total intestinal TG production in CD36KO mice during postprandial state. The enhanced intestinal TG production may consequently cause postprandial hyperlipidemia in subjects with CD36-deficiency.

It is also reported that CD36 ablation causes lipid accumulation in the enterocytes of the upper villi of the proximal small intestine after an acute lipids load and with a HFD treatment, indicating the lipid homeostasis is disrupted in intestinal enterocytes of CD36-null mice (Drover et al., 2005; Masuda et al., 2009). Since the lipids uptake defects in the proximal intestine of CD36KO mice, the accumulated lipids in intestine of CD36-null mice may be attributed to enhanced *de novo* lipids synthesis and/or decreased lipids transport/secretion into the lymph.

Consistently, the mRNA level of fatty acid synthase (FAS), a key enzyme involved in the biosynthesis of FFA, is also significantly increased in the intestinal epithelium of CD36KO mice, both in the fasting state and postprandial states, when compared with WT mice. The mRNA level of stearoyl-CoA desaturase 1 (SCD1), a lipogenic enzyme in the biosynthesis of monounsaturated fatty acids [mainly oleate (C18:1) and palmitoleate (C16:1)], is increased significantly in CD36KO mice in the postprandial state (Masuda et al., 2009). On the other hand, it is suggested that CD36 is necessary for inducing the expression of apoB48, microsomal triglyceride-transfer protein (MTP) in response to LCFA loading, and the formation and maturation of pre-chylomicron (pre-CM) transport vesicle, which are key steps of CM formation/transport (Tran et al., 2011). The average size of the lymph lipoprotein particles of CD36KO mice was significantly smaller than that of WT mice in the lipid feeding state. The lymphatic apoB amounts are also lower in CD36KO mice than WT mice during both fasting and postprandial states (Masuda et al., 2009). These data support that CD36 deficiency promotes *de novo* lipid synthesis and blunt CM formation/transport in the intestine. However, it remains unclear exactly how CD36 might regulate intestinal *de novo* lipids synthesis and CM production (Figure 1).

Notably, in addition to regulating LCFA transport into the lymphatic vessel under physiologic conditions, CD36 may contribute to abnormal LCFA absorption under pathological conditions. In patients with liver cirrhosis, it is suggested that the increased expression of glycosylated CD36 in intestinal endothelial cells may promote alternative LCFA transport into blood capillary vessels, rather than into lymphatic vessels because of the backpressure of these vessels as a result of portal hypertension (Yamamoto et al., 2012).

### The Regulation of Lipids on Intestinal CD36

CD36 has been identified as a receptor for various nutrients and its products, for example, advanced glycation end products (AGEs), modified-lipoproteins and LCFA. In turn, multiple nutrients and its derivatives also modulate CD36 expression and function (Shi et al., 2019).

In patients with hyperglycemia and/or hyperlipidemia, the CD36 expression is significantly increased in vascular lesions and kidneys compared with control subjects (Shu et al., 2020). In duodenal mucosa biopsies from human, the CD36 expression is also positively correlated with increasing body mass index (BMI), suggesting the dysregulation of CD36 responses to fat in obesity (Little et al., 2014). In addition, HFD significantly increased CD36 expression in the jejunum of mice (Lynes et al., 2011).

Fatty acid and its products promote CD36 transcription by activating NF-KappaB pathway or peroxisome proliferator-activated receptors (PPARs) or TR4 pathway (Xie et al., 2009; Jiang et al., 2019). The ox-LDL promote CD36 transcription by activating PPARs signaling pathway contributing to foam cell formation in macrophages (Gautam and Banerjee, 2011). Glucose also promotes CD36 translation via increasing ribosomal reinitiation following enhanced translation of an ORF downstream of the CD36 5′-untranslated region (UTR) (Griffin et al., 2001).

In addition, recent studies indicate that nutrients regulate CD36 expression and translocation at the post-translational levels. FA increases poly-ubiquitination of CD36, contributing to reduce of CD36 protein levels and decrease of cellular fatty acid uptake in C2C12 myotubes and CHO cells (Tran et al., 2011). Interestingly, treatment of FA in cardiomyocytes promotes the translocation of CD36 from endosomes to the sarcolemma by activating vacuolar-type H^+^-ATPase, rather than alter myocellular protein levels of CD36 (Liu et al., 2017). FA supply can increase the O-linked N-acetylglucosamine (O-GlcNAc) modification of CD36 via hexosamine biosynthesis pathway, resulting in an increase of CD36 expression on cell membranes of gastric cancer cells (Jiang et al., 2019). We have previously demonstrated that FA and HFD promote CD36 palmitoylation and distribution in the plasma membranes of hepatocytes, contributing to NAFLD development and progression (Zhao et al., 2018b). Recent studies also demonstrate the importance of FA in regulating the internalization of CD36, which facilitates the transport of FA into adipocytes (Hao et al., 2020). During this process, binding of FA to CD36 activates its downstream kinase Lyn, which promotes the Tyr91 phosphorylation and inactivation of palmitoyl acyltransferase DHHC5, the palmitoyl acyltransferase of CD36 (Wang et al., 2019). CD36 then gets depalmitoylated by acyl-protein thioesterase 1 (APT1) and recruits another tyrosine kinase SYK to phosphorylate c-Jun N-terminal kinase (JNK) and VAVs to initiate delivery of FAs into cells. These observations underscore the notion that the regulation of FA on CD36 expression/function is tissue-specific and/or via different mechanisms.

### Conclusion

With the recognition of the gut-liver axis, the gut-pancreas axis, and the gut-brain axis, the key roles of the intestine in the maintenance of energy homeostasis and diet-related metabolic disorders have been well-accepted. Numerous studies have reported that CD36 is closely related to the development of Mets, atherosclerosis, NAFLD and diabetes. Although the intestinal CD36 is involved in mediating dietary lipids uptake, it seems to play an essential role in the transduction of lipid signals into epithelial cells regulating *de novo* lipid synthesis, lipids transport and gastrointestinal hormones production/secretion in response to dietary lipid loading. However, much knowledge of CD36 in the intestine is now from humans and animal models where CD36 is less functional or absent systemically. Thus, the intestinal-specific CD36 knockout models are needed to validate the importance of intestinal CD36 in sensing dietary lipids and regulating lipid homeostasis in the future. Moreover, it remains unclear how CD36 regulates intestinal lipoprotein/hormone production and secretion in the fasting and postprandial states. It is also unclear how fat loading in the intestine regulates CD36 expression and post-translational modifications (Figure 1). Further studies are needed to demonstrate the complex interactions between intestinal CD36 and dietary lipids.

## Author Contributions

LZ, YuL, QD, and YaL wrote the manuscript. YC and XR revised the manuscript. All authors contributed to the article and approved the submitted version.

## Conflict of Interest

The authors declare that the research was conducted in the absence of any commercial or financial relationships that could be construed as a potential conflict of interest.

## Footnotes

1 https://www.ncbi.nlm.nih.gov/gene/948
